# The effect of linking community health centers to a state-level smoker's quitline on rates of cessation assistance

**DOI:** 10.1186/1472-6963-10-25

**Published:** 2010-01-25

**Authors:** Donna Shelley, Jennifer Cantrell

**Affiliations:** 1 Department of Cariology and Comprehensive Care and Department of General Internal Medicine, New York University College of Dentistry, New York, NY, USA; 2 Public Health Solutions, National Development Research Institutes, Inc., Behavioral Science Training Program, New York, NY, USA

## Abstract

**Background:**

Smoking cessation quitlines are an effective yet largely untapped resource for clinician referrals. The aim of this study was to assess the effect of a fax referral system that links community health centers (CHCs) with the New York State Quitline on rates of provider cessation assistance.

**Methods:**

This study was conducted in four CHCs using a quasi experimental study design. Two comparison sites offered usual care (expanded vital sign chart stamp that prompted providers to ask about tobacco use, advice smokers to quit, assess readiness, and offer assistance (4As)) and two intervention sites received the chart stamp plus an office-based fax referral link to the New York State Quitline. The fax referral system links patients to a free proactive telephone counseling service. Provider adherence to the 4 As was assessed with 263 pre and 165 post cross sectional patient exit interviews at all four sites.

**Results:**

Adherence to the 4As increased significantly over time in the intervention sites with no change from baseline in the comparison sites. Intervention sites were 2.4 (p < .008) times more likely to provide referrals to the state Quitline over time than the comparison sites and 1.8 (p < .001) times more likely to offer medication counseling and/or a prescription.

**Conclusions:**

Referral links between CHCs and state level telephone quitlines may facilitate the provision of cessation assistance by offering clinicians a practical method for referring smokers to this effective service. Further studies are needed to confirm the efficacy of fax referral systems and to identify implementation strategies that work to facilitate the utilization of these systems across a wide range of clinical settings.

## Background

According to the 2008 Public Health Service (PHS) guideline *Treating Tobacco Use and Dependence *there is strong evidence that screening, brief intervention and pharmacotherapy can produce significant and sustained reductions in tobacco use [1]. However, clinicians find it challenging to deliver all of the recommended 5As (ask, advise, assess, assist, arrange) in the context of a busy practice [2]. Strategies to assist providers in streamlining treatment, such as including smoking as a vital sign, increases screening rates but does not increase cessation assistance as a standalone intervention [3-6]. Reorganizing the practice environment to help clinicians delegate the time consuming steps of counseling and arranging follow-up may improve rates of treatment delivery and enhance the level of support smokers receive. One way this can be accomplished is by linking health care settings with statewide telephone counseling programs.

Telephone quitlines have proven effective in increasing smoking cessation and are now available in all 50 states in the US through a national network of tobacco quitlines [1]. All but one state quitline allows providers to refer patients through fax referral programs [7]. The "Fax-to-Quit" program, as it is referred to in New York State, aims to simplify the referral process by offering clinical practices a method to link patients to a free proactive telephone counseling service using a fax referral form. In a proactive approach the counselor initiates the call, in contrast to reactive telephone counseling in which the patient initiates contact. A recent meta analysis found that smokers who receive proactive counseling are 56% more likely to quit compared with self help [8].

Recent studies suggest that connecting clinicians to telephone tobacco quitline counselors through fax referral systems is feasible and may induce providers to intervene more consistently with their patients who smoke [9-17]. However this service has not been well studied in community health centers (CHCs) that serve low income minority populations. The primary purpose of this pilot study was to examine the effect of implementing the fax-to-quit system plus an expanded vital sign chart stamp that prompted providers to offer the first 4As compared to the vital sign chart stamp alone on the level of cessation support smokers received.

## Methods

### Clinical settings and study population

From September 2006 through November 2007, we conducted a quasi-experimental study in two intervention and two comparison CHCs located within the New York Presbyterian Hospital Ambulatory Care Research Network (ACRN), a practice-based research network of 12 CHCs. These clinics serve a primarily low-income Latino immigrant population. At the time of the study the clinics were using a uniform paper chart system.

#### Usual care

Prior to the study, as part of a quality improvement (QI) initiative to disseminate tobacco use treatment guidelines, all CHCs within the ACRN implemented an expanded vital sign chart stamp that prompted providers to ask patients about tobacco use, advise them to quit, assess readiness to quit and offer assistance (4As) (Additional file 1). The prompt to provide assistance was divided into two components on the chart stamp: prescription given and referral made. This approach was meant to simplify the documentation process and operationalize the 4th A, Assist, to make it clear that referral and prescription were the two primary options available to the provider and patient.

After implementation of the new chart system, all providers attended a 60 minute physician led presentation on current evidence based practice guidelines for treating tobacco dependence and systems level changes to support identification and referral of smokers for cessation treatment. At the conclusion of this visit, each practice received a tool kit consisting of patient education materials and provider materials (e.g. pharmacotherapy guide) and wallet cards with the quitline number.

#### Intervention

The intervention was comprised of four components: 1) usual care plus the fax referral system that linked smokers to the New York State Quitline for proactive tailored counseling, 2) a 30 minute training for physicians, nurses and medical assistants on how to use the Fax-to-Quit program, 3) two site visits from research staff that involved meeting with clinic staff to elicit any barriers to implementation, provide additional materials and offer further educational information as needed, and 4) provider feedback on their adherence to the 4As and use of the Fax-to-Quit program compared with other providers in their clinical site. Feedback data was embedded in two separate emails sent during the four month intervention period.

The New York State Quitline service includes proactive telephone calls with mailing of self help material, free nicotine replacement therapy for those who qualify and referrals to local treatment programs. The quitline faxes a report back to the provider describing the treatment plan. Providers are also notified if the patient cannot be reached. The quitline makes up to five attempts to contact patients.

### Data collection

Provider performance was assessed with 263 pre and 165 post intervention cross sectional patient exit interviews. Trained research assistants (RA's) screened each patient in the waiting room before their clinic visit to assess eligibility for the study and to obtain consent. Patients were eligible if they were aged 18 years or older, a current smoker, and the visit was with a primary care provider (excluding Obstetrics and Gynecology). Eligible patients were asked to complete the in person interview after their visit. The screening survey included questions about fruit and vegetable intake and daily exercise. Patients were not told that the research study was related to tobacco use before seeing their doctor or nurse. The study protocol was reviewed and approved by the Institutional Review Boards of Columbia University and New York University.

### Measures

#### Patient characteristics

The exit interview survey collected information on patient demographics (age, gender, education, race/ethnicity and health insurance), the nature of the visit (initial or routine, scheduled or walk in), tobacco use behavior (number of cigarettes smoked and if smokers smoked every day or some days) and readiness to quit in next 30 days.

#### Adherence to 4As

Exit interview surveys also collected data on provider adherence to the 4As. Questions assessed whether patients were screened for tobacco use, offered advice to quit, assessed for readiness to quit, offered a referral to the Fax-to-Quit program, accepted the referral, and if they were offered counseling about medication options and/or a prescription (e.g., During your visit today, did a health care provider advise you to quit smoking or using tobacco?). The two main outcome measures included: 1) patient self report of being offered a referral to the Fax-to-Quit program from a clinician or staff; and 2) provision of medication education and/or prescription.

### Data Analysis

Descriptive statistics were used to obtain rates of adherence to guideline recommendations to ask, advise, assess, refer and prescribe both within groups over time and between groups. We conducted Chi-square tests to compare differences in patient characteristics between intervention and comparison groups and to examine changes in patient characteristics over time within groups. We also conducted two-group mean comparison t-tests to analyze differences within groups and between groups at baseline and follow-up. Fixed effect logistics regressions were used to predict whether the main outcome variables changed with time in the intervention and comparison settings, controlling for clustering at the clinic level. We employed univariate regressions to examine patient characteristics that were associated with Fax-to-Quit referrals and medication education at baseline across all clinics. Characteristics significantly associated with the outcomes were controlled for in the final multiple regression analyses. All statistical analyses were performed using Stata 10.0 software [18].

## Results

### Sample

Table 1 shows pooled data on demographic characteristics and smoking behavior for the intervention and comparison sites at baseline and follow-up. Among the total sample of 460 smokers, 85% were Hispanic and 65% female. Sixty-two percent were over the age of 50 and 82% had Medicaid. Twenty percent of all respondents smoked over 15 cigarettes per day (CPD). Over 60% of smokers reported that they were ready to quit in the next 30 days. Patient demographics and smoking behaviors did not differ overall or within sites between the two time periods, with the exception of smoker readiness to quit, which decreased equivalently across all sites. Patient smoking behaviors did not differ between the sites at either time period.

**Table 1:** Demographic and smoking characteristics for the pooled sample at baseline and follow-up

Characteristics	Baseline (n = 258)	Follow-up (n = 202)	p-value
Male	36.4%	33.2%	.47
Age in years			.75
18-49	38.5%	40.0%	
50+	61.5%	60.0%	
Race/ethnicity			.10
White	2.3%	5.0%	
Black	12.8%	7.9%	
Latino	84.5%	85.6%	
Other	.39%	1.5%	
Less than high school education	55.4%	59.9%	.34
Health care insurance			
Private insurance	1.9%	4.0%	.30
Medicare	10.5%	9.4%	
Medicaid	80.2%	81.7%	
No coverage	2.3%	3.0%	
Smoke frequency			.19
Some days	22.9%	17.8%	
Everyday	77.1%	82.2%	
Smoke 15 or more cigarettes per day	17.8%	22.8%	.19
Smoke within 30 minutes of waking up	41.1%	46.3%	.27
Ever attempted to quit	73.7%	70.0%	.83
Ready to quit in next 30 days	76.0%	45.1%	.00

*Note: *Chi square test was used to test the differences between baseline and follow-up groups. Missing rates were negligible or zero for all variables.

### Screening, advice and assessment

At baseline, intervention and comparison sites did not differ significantly in their adherence to the recommended first 3 As (ask, advise and assess). Over 90% of patients were asked about tobacco use, over 80% of smokers were advised to quit and more than 65% were assessed in terms of readiness to quit across all sites (Table 2). Among intervention sites, screening and assessment rates at follow-up increased compared to baseline by 7.6% (91% to 98%) (p = .01) and 23% (65% to 80%) (p = .003), respectively, but remained unchanged in comparison sites.

**Table 2:** Provider adherence to tobacco use treatment guidelines based on patient exit interviews at baseline and follow-up

Tobacco Assessment Measures	Intervention Group			Comparison Group		
	**Baseline** **(n = 156)**	**Follow-up (n = 138)**	**p-value**	**Baseline** **(n = 102)**	**Follow-up** **(n = 64)**	**p-value**

Ask	91%	98%	.01	94%	91%	.39
Advise	83%	91%	.07	83%	87%	.49
Assess	65%	80%	.003	71%	78%	.34
Refer to Fax-to-Quit	17%	35%	.000	44%	37%	.40
Discuss medications	49%	65%	.003	60%	63%	.72

*Note: *The analyses were conducted using a two-group mean comparison t-test. The p-values reflect differences within groups between baseline and follow-up.

### Referrals

Baseline rates of assistance (i.e., referred to Quitline and discussed medication) were higher in the comparison group but this difference did not reach significance. In intervention sites, referrals to the Quitline increased from 17% to 35% (p = .001) and counseling about medication increased from 49% to 65% (p = .005). In contrast, among comparison sites, referrals to the New York State Quitline did not change significantly between the baseline and follow-up period. Data received from the New York State Quitline indicated that they reached 41% of all patients referred from study sites during the intervention period.

### Regression analysis

The intervention sites were 2.4 times more likely to provide referrals to the state Quitline over time than the comparison sites and 1.8 times more likely to offer medication counseling and or a prescription (Table 3). Older smokers, those with a higher level of dependence and smokers who were ready to quit in the next 30 days were more likely to receive education on pharmacotherapy.

**Table 3:** Logistic regressions analyzing impact of intervention on offer of referrals and provision of a smoking cessation prescription or education related to pharmacotherapy

Dependent variables	Odds Ratio	p-value (95%CI)
^a^Referral to NYS Quitline (n = 458) Interaction (time and intervention)	2.4	.008 (1.25, 4.63)
^b^Provided pharmacotherapy education or prescription (n = 453) Interaction (time and intervention)	1.8	.001 (1.26, 2.63)

^a^Logistic regression with offer of referral to Quitline coded as 1. Adjusted for time to smoke upon waking.

^b^Logistic regression medication education coded as 1. Adjusted for time to smoke, smoking frequency (everyday or some days), readiness to quit and age.

## Discussion

Tobacco quitlines are an effective yet largely untapped referral resource for health care providers who may not have the time or resources to offer onsite smoking cessation counseling. In this study, a fax referral system combined with an expanded vital sign chart prompt was associated with higher rates of provider initiated assistance and referral to the Quitline compared with the chart prompts alone. These results suggest that Fax-to-Quit programs can be implemented in busy CHC practices, and similar to previous research, can facilitate cessation assistance through referrals to state-level telephone counseling services [9-17].

The high rates of baseline screening and advice to quit offered by clinicians in all study sites was likely related to the expanded vital sign chart stamp which was implemented three months prior to the start of this study. The Ambulatory Care Network QI committee's decision to use the enhanced chart stamp was informed by research demonstrating that smoking status vital sign chart stamps that only prompt providers to screen for tobacco use do not consistently increase provider delivered advice to quit or cessation assistance [3-6]. However, our results suggest that even a more elaborate chart stamp, which prompts providers to refer and prescribe, may not be adequate to trigger clinician action without an accompanying referral system in place that is fully operationalized (i.e. staff training, technical assistance, staff roles defined in relation to the referral system, faxing process in place). Comparison sites were aware of the Quitline and the fax referral system, but they only received preprinted referral pads with the Quitline telephone number which they were advised to give to smokers who were ready to quit. In contrast, intervention sites received additional training and ongoing technical assistance that was specifically related to implementing and using the fax referral system.

Several evaluations of state level quitline referral systems have noted the need for supportive infrastructure including a site champion, training, technical assistance and feedback to facilitate the interaction between clinicians and the centralized quitline, increase adoption and ensure successful implementation [10,16-19]. Intervention clinics received several of these components, while comparison sites were familiar with the Fax-to-Quit program but did not receive the support that may be needed to fully utilize the service. Simply knowing about the program may not be enough to promote consistent and sustainable referrals. Post intervention qualitative interviews with physicians and staff, the results of which are reported in another publication, support this premise [20]. Health care providers and staff in intervention sites described numerous benefits of the chart stamp and Fax-to-Quit systems but pointed out that those new systems, even ones that will ultimately simplify care processes, can initially interrupt workflow and must overcome organizational barriers to ensure full integration and sustainability [20].

Of note, this study coincided with implementation of a well funded aggressive statewide tobacco control program that included funding of 19 cessation centers across the state. Initiated in 2004, the centers were established to implement and disseminate the PHS tobacco use treatment guidelines in a wide range of health care settings (inpatient and outpatient). Specific emphasis was made on increasing clinician referrals to the New York State Quitline via the Fax-to-Quit program. Statewide cessation center activities have been associated with a 132% increase in fax referrals from providers over the past 5 years. Referrals increased from 4,215 in 2005 to a projected 9,796 for 2009. Moreover, health care settings that have received the most attention in terms of technical assistance and implementation support have the highest rates of referrals [[10], NYSDOH Quitline, 2009 unpublished data].

The Fax-to-Quit program offers several advantages to patients and providers, including easy access to a free, evidence-based counseling program regardless of insurance status, the ability to serve diverse multilingual populations, links to local cessation programs that providers may not be aware of, and in many states, free pharmacotherapy [6,9,16,21]. Enhancing fax referrals may also increase overall engagement of providers in cessation activities. Although changing practice patterns related to prescribing was not a specific target of the intervention, we found that intervention sites were almost twice as likely to offer assistance with smoking cessation medications. A recent survey of Swedish General Practitioners (GPs) also found that GPs who referred to a quitline were more likely to be active in other smoking cessation activities. Provider knowledge and use of the quitline may have an additional positive effect on overall engagement in treating tobacco use [22].

One of the drawbacks of the proactive program is low contact rates. Studies of fax referral programs in health care settings report quitline contact rates ranging from approximately 40-70% [10-16,23,24]. During the intervention period only 41% of patients were reached, which is lower than the average NYSDOH contact rate of 53% (NYS DOH unpublished, 2009). The low contact rates in this study may be related to the demographic characteristics of this population. Mahabee-Gittens reported similar contact rates (46%) in a study of low income, minority parents referred for proactive quitline counseling in a pediatric emergency room [14].

Improving connection rates may require additional staff and provider training to ensure that patients are assessed properly. In addition, patient education prior to referral may help normalize the service, clarify expectations and overcome resistance that patients may not be willing to express to their doctor [20]. One of the providers interviewed for this study reported connecting patients to the Quitline while they were still in the office. Assisting patients through care coordinators or other staff during or soon after the visit may improve connection rates and increase referrals [24]. An additional reason to address this issue is the potentially negative impact of low patient contact rates on provider referrals. Providers may be discouraged from continuing to refer patients to a program that cannot consistently reach their patients. Collaboration with providers and quitlines is needed to develop new strategies for improving patient connections with proactive quitline counseling [16].

Ask-advise-refer and ask and act models that integrate the 5As into an abbreviated intervention supported by quitline fax referral or web-based programs have been promoted as a method to simplify adoption of guideline recommended tobacco use treatment in primary care [25-29]. The Fax-to-Quit program provides a simple method for implementing this more streamlined approach. However, there are several questions regarding how to best integrate quitline-practice linkages and the impact of these programs on provider practice patterns and patient outcomes. For example, are providers less likely to engage patients and provide follow-up if they assume the quitline will take over this responsibility, and does this reduce the impact on cessation outcomes compared to the 5As approach [21,23,30,31]? Borland *et al*. found that the option for referring did not result in GPs providing less effective smoking cessation counseling within the consultation, but this requires further study [30]. Second, what are the barriers to adopting and maintaining fax or email referral systems in busy clinical settings? Further studies are needed to identify the specific implementation strategies and systems level approaches that work to facilitate routine delivery of tobacco cessation services and specifically, routine referral to state-level quitlines.

A number of limitations require consideration. The study sample was small and the CHCs were not randomized. The CHCs practice, however, under the same administrative umbrella at one academic institution and serve the same patient population. Moreover, an analysis of patient and provider characteristics demonstrated no baseline differences between sites. There were also no differential changes in patient characteristics at the baseline and follow-up time periods. However, our findings require replication. Another potential limitation was the dependence on patient self reports. We collected faxed forms at the intervention sites only, which confirmed patient reports of fax referrals, but we did not have the same data from the comparison sites.

## Conclusion

State quitlines provide an important resource for health care providers and patients. Smokers in every state in the U.S. have free access to telephone quitlines through 1-800-QUIT-NOW and the fax referrals system is available in 49 of 50 states [7]. These programs have the potential to be replicated across a multitude of health care settings and may be a key strategy for facilitating routine cessation assistance and referrals into routine primary care.

## Competing interests

The authors declare that they have no competing interests.

## Authors' contributions

DS designed the study and survey tools, assisted with data analysis and wrote the manuscript. JC completed the literature review for the manuscript, assisted with survey design, analyzed the survey data, and wrote and edited portions of the manuscript. JC and DS provided substantial subsequent contributions. Both authors read and approved the final manuscript.

## Pre-publication history

The pre-publication history for this paper can be accessed here:

http://www.biomedcentral.com/1472-6963/10/25/prepub

## Supplementary Material

Additional file 1 **Figure S1**. Expanded vital sign chart stamp. Click here for file
