# Supplementary material for: The effect of linking community health centers to a state-level smoker's quitline on rates of cessation assistance
Source: BMC Health Serv Res. 2010 Jan 25;10:25. doi: 10.1186/1472-6963-10-25 (PMC2823740; doi:10.1186/1472-6963-10-25)
Supplement: Additional file 1 — Figure S1. Expanded vital sign chart stamp. [file 1472-6963-10-25-S1.DOC]

BP: __________ Weight: _______

Ht: _______ BMI:_____

Tobacco Use: Yes No Former

Advise to quit: Yes No

Ready to quit? Yes No

Rx given: Yes No

Referral made: Yes No
